# Respiratory Microbial Co-infection With SARS-CoV-2

**DOI:** 10.3389/fmicb.2020.02079

**Published:** 2020-08-25

**Authors:** Bill W. Massey, Karuna Jayathilake, Herbert Y. Meltzer

**Affiliations:** ^1^Vikor Scientific LLC, Charleston, South Carolina, SC, United States; ^2^Department of Pharmacology and Interdisciplinary Toxicology, University of Arkansas for Medical Sciences, Little Rock, AR, United States; ^3^Department of Psychiatry and Behavioral Sciences, Northwestern Feinberg School of Medicine, Chicago, IL, United States

**Keywords:** SARS-CoV-2, COVID-19, respiratory co-infection, *K. pneumoniae*, *M. catarrhalis*, nursing home, age, race

## Abstract

Co-infection with additional pathogens is a well-known feature of pandemics. We determined the prevalence and type of a wide variety of respiratory pathogens in 12,075 United States subjects tested for SARS-CoV-2 infection in March and April 2020. Infections with other respiratory pathogens, which on their own produce at least some SARS-CoV-2 symptoms including mortality, were present in both SARS-CoV-2 + and SARS-CoV-2- subjects. Non-SARS-CoV-2 infection rates were significantly higher in SARS-CoV-2 + (86%) patients than SARS-CoV-2– patients (76%) (*p* < 0.0001). Among the co-pathogens present in both subject groups were *K. pneumoniae* and *M. catarrhalis* which can produce serious respiratory illness on their own, Advanced age and nursing home status were associated with higher SARS-CoV-2 + and co-infection rates. Testing for the presence of co-pathogens going forward will assist in the diagnosis and optimal treatment of suspected SARS-CoV-2 respiratory infections in the current pandemic.

## Introduction

SARS-CoV-2 infected individuals present with fever, aches, headache, cough, runny nose, nasal congestion, coagulation-induced vascular pathology, and fatigue (World Health Organization, 2020). An estimated 5 to 80% of infected individuals are asymptomatic, while 3 to 4% will die despite current approaches to treatment (Nishiura et al., 2020). These symptoms are also present in other, sometimes fatal respiratory infections, e.g., *K. pneumonia*, and *M. catarrhalis* (Cox et al., 2020). Patients with primary SARS-CoV-2 or other respiratory infections may have increased susceptibility to a secondary respiratory infection, with the probable result of exacerbations of disease severity should both be present (Cox et al., 2020). It is likely that co-infection with some pathogens will lead to greater severity of illness and complicate treatments which address only SARS-CoV-2 or the co-pathogen (Cox et al., 2020). There is limited information about respiratory co-infection in SARS-CoV-2 subjects.

A few studies have reported co-infection rates in SARS-CoV-2 infected patients. A United States study of 116 SARS-CoV-2 + subjects found that 20% were co-infected with other pathogens (Kim et al., 2020). A recent online study in a metropolitan New York population, sampled in March 2020, examined a limited number of relatively benign pathogens in 1,204 SARS-CoV-2 + cases and found coinfection in only 36 SARS-CoV-2 + patients (3%), while 111/845 (13.1%) of SARS-CoV-2- patients were co-infected (Nowak et al., 2020). The study tested for Influenza A and B, respiratory syncytial virus, other coronaviruses, rhinovirus, human metapneumovirus, adenovirus and parainfluenza viruses. Bacterial pathogens were not tested in this study. The most common detected pathogen in SARS-CoV-2- patients was rhinovirus (5.93%), whereas in SARS-CoV-2 + patients the most common co-pathogen were non-SARS-CoV-2 coronaviridae viruses (Nowak et al., 2020). Of note, only 13.4% of the 1,204 SARS-CoV-2 + and 11.4% of the 7,418 SARS-CoV-2- patients were tested for co-pathogens. The basis for sample selection was not disclosed and may not be representative of the population at risk.

Studies examining coinfection rate in SARS-CoV-2 infected patients have revealed a wide variance in coinfection rates, ranging from 0.6 to 50% (Lai et al., 2020). The observed coinfected pathogens have been viral (e.g., influenza, rhinovirus, parainfluenza, metapneumovirus, HIV), bacterial (e.g., *Staphylococcus aureus*, *Streptococcus pneumoniae*, *Klebsiella pneumoniae*, *Mycoplasma pneumoniae*, *Chlamydia pneumoniae*, *Legionella pneumophila*), and fungal (e.g., *Candida* species, *Aspergillus flavus*) (Lai et al., 2020). The most common coinfected pathogens varied from study to study, and this variability may be due to geographic factors, differences in the breadth of organisms tested, or disparate patient demographic factors (Lai et al., 2020). In addition, several case studies of coinfection in SARS-CoV-2 patients have been reported and include coinfection with influenza A (D’Ardes et al., 2020; Konda et al., 2020; Wu et al., 2020).

As an indication of the importance of this issue, bacterial co-infection was reported in half of the deceased patients in a retrospective study of SARS-CoV-2 from Wuhan, China (Zhou et al., 2020). This may be due to increased risk of pneumonia, the primary cause of SARS-CoV-2 deaths in co-infected individuals (Gao et al., 2020). The fatal pneumonia cases may result from an excessive immune reaction to SARS-CoV-2 called “cytokine storm,” elicited by the response to multiple pathogens (Zhou et al., 2020). The extent to which one type of infection affects the susceptibility to the other in SARS-CoV-2 is unknown. Respiratory co-infections were reported to be major contributors to morbidity and mortality in previous viral respiratory epidemics, such as the influenza pandemics of 1918 and 2009 (Morens et al., 2008; MacIntyre et al., 2018). During the 2009 influenza pandemic, bacterial infections were found in almost 25% of patients and were associated with higher rates of intensive care unit admissions and death (MacIntyre et al., 2018).

In the present study, we present data on the prevalence of SARS-CoV-2 and other bacterial, viral and fungal respiratory pathogens in samples taken from over 12,000 United States symptomatic subjects tested for the presence of SARS-CoV-2 in a manner which permitted assessment of the presence of co-pathogens as well as SARS-CoV-2.

## Materials and Methods

### Subjects

The data were obtained from 12,075 subjects from whom nasopharyngeal swabs were collected with the principal aim to detect presence or absence of SARS-CoV-2 infection. Subjects consent to use de-identified data for research purposes was obtained with the test requisition form. These samples were collected and submitted to Vikor Scientific, a CLIA-Certified and CAP-Accredited clinical laboratory located in Charleston, South Carolina between March 25, 2020 and April 22, 2020. The samples were collected from multiple types of health facilities throughout the United States and shipped by overnight mail for analysis. PCR analyses were conducted the day of arrival at the lab to identify SARS-CoV-2 and a panel of 38 other bacterial, fungal, and viral respiratory pathogens.

### Laboratory Analytical Methods

Pathogen testing was performed by real time PCR amplification to detect presence of tested pathogens by amplifying genomic DNA/RNA. The microorganisms tested are listed in Table 1. Amplification and detection were performed using an Applied Biosystems™ QuantStudio™ 5, 12K Flex real-time PCR system (Catalog # A34322; Thermo Fisher Scientific, Waltham, MA, United States). Real-time PCR amplification was performed using TaqMan™ assays from Thermo Fisher Scientific consisting of two PCR primers and one fluorescently labeled probe which hybridizes to the target organism’s genomic nucleic acid. Semi-quantitative determination of pathogen load for co-infected pathogens was derived from *C*_t_ values and presented as number of cells per mL for bacterial pathogens (e.g., 1 × 10^5^ cells/mL) and as number of viral copies per mL for viral pathogens (e.g., 1 × 105 viral copies/mL).

**TABLE 1:** Respiratory pathogens in screening panel.

Adenovirus 1 and 2 Alpha	Respiratory Syncytial Virus A and B
Adenovirus 1 and 2 Beta	Varicella zoster virus (HHV3)
Coronavirus HKU1	*Bordetella* (PAN)
Coronavirus NL63	*Bordetella pertussis*
Coronavirus OC43	*Chlamydophila pneumoniae*
SARS-CoV-2	*Coxiella burnetii*
Cytomegalovirus (HHV5)	*Haemophilus influenzae*
Enterovirus A/B/C	*Klebsiella pneumoniae*
Epidemic Parotitis (Mumps)	*Legionella pneumophila*
Epstein-Barr virus (HHV4)	*Moraxella catarrhalis*
Human Bocavirus (HBoV)	Mycobacterium avium complex (MAC)
Human Herpesvirus (HHV6)	*Mycoplasma pneumonia*
Human Metapneumovirus	*Pseudomonas aeruginosa*
Influenza A virus (Pan)	*Staphylococcus aureus*
Influenza B virus (Pan)	*Streptococcus agalactiae (group B)*
Parainfluenza 1	*Streptococcus pneumoniae*
Parainfluenza 2	*Streptococcus pyogenes*
Parainfluenza 3	*Aspergillus fumigatus*
Parainfluenza 4	
Parechovirus	

SARS-CoV-2 testing was performed using the TaqPath™ COVID-19 Combo Kit (Catalog # CCU002; Thermo Fisher). The assay detected three SARS-CoV-2-specific gene targets: ORF1ab; S Protein; and N Protein. Full validation studies established a Limit of Detection 95% (LOD_95_) of 7.8 copies/uL. Linear dynamic range sensitivity over a positive control range of 1–1,000 copies/uL was demonstrated at *R*^2^ > 99%. No cross-reactivity was seen in the multi-target plasmid control containing common respiratory pathogens nor to any respiratory pathogens tested using Thermo Fisher TaqMan™ assays. Detection of the SARS-CoV-2-specific gene targets were each considered positive at *C*_t_ values < 37, inconclusive at *C*_t_ values 37 to 39, and negative at *C*_t_ values > 40. Patients were considered positive for SARS-CoV-2 if two or more of the SARS-CoV-2 genes were detected.

Analytical accuracy was evaluated using the control material contained in the Thermo Fisher TaqPath™ Covid-19 Combo Kit (Catalog # A47533) for each of the 3 target sequences for gene assays ORF1ab, S protein, and N protein. Controls with concentrations of 1X, 2X, and 3X LOD_95_ were used and run in triplicate. A positive/negative amplification status was used to satisfy the C_t_ criteria and considered successful when the “actual” call concurred with the “expected” call. Analytical accuracy was evaluated based on the comparison of “expected” call and “actual” call for RNA spiked samples from 1X-6X LOD_95_ and the Non-Template Control (water without nucleic acid). Results based upon concordance between “actual” and “expected” calls were 100% concordant for all 3 gene targets of SARS-CoV-2.

Co-infected pathogens were tested using Respira-ID™ (Vikor Scientific, Charleston, SC, United States), a custom respiratory pathogen panel consisting of TaqMan™ assays on a preloaded TaqMan™ array card. The list of tested respiratory pathogens is shown in Table 1. Full validation studies established a LOD_95_ of 50 copies/PreAmpReaction of input, with only the Herpesvirus 6 assay showing an LOD_95_ at 250 copies/PreAmpReaction. Linear dynamic range sensitivity over a positive control range of 4 logs was demonstrated with *R*^2^ values > 0.98. Potential cross reactivity was evaluated using well-characterized, normalized genomic DNA pools, with specificity across all assays shown to be 99.89%. Analytical accuracy was evaluated based on comparison to well-characterized, normalized genomic DNA and multi-target plasmid controls and found to be 100%.

### Data Acquisition

A data query engine was developed and used to mine data from the laboratory information management system (OvDx, v1.33; Ovation.io, Inc., Cambridge, MA, United States). The data query engine extracted all available information on all tested patients, including all test results and all patient information provided on the test requisition submitted by the ordering physician. These data were organized in a spreadsheet format for importation into the SAS™ statistical software used for analyses. The SARS-CoV-2 and co-infection data were then analyzed for the frequency and pattern of co-detection of other pathogens. Patients were grouped by health facility type, age and gender for additional analysis. Patients were grouped into the following age categories: >62 years of age (elderly), 19–61 years of age (adult), and < 19 years of age (young). The source of the samples, age, and gender of the samples are provided in Table 2. No samples were collected from incarcerated individuals.

**TABLE 2:** Relation of gender, race, age group and residential status to SARS-CoV-2 ± subjects.

**Variable**	**Category**	**SARS-CoV-2 +**		**SARS-CoV-2 -**		**Total**	***p*-value**
		**N**	**%**	**N**	**%**	**N**	
Gender	Male	470	13.4	3036	86.6	3506	NS
	Female	986	13.5	6324	86.5	7310	
Race	Caucasian	652	15.2	3652	84.9	4304	NS
	Afro. American	78	12.6	541	87.4	619	
Age Group	Age < 62	471	8.4	5145	91.6	5616	*p* <0.0001
	Age ≥62	1206	18.8	5208	81.2	6414	
Testing	Resident	953	20.3	3748	79.7	4701	*p* <0.0001
Location	Outpatient	737	10.0	6637	90.0	7374	

### Data Analyses

The *t*-test for independent group comparison was used to compare age of the study subjects across gender, SARS-CoV-2 ± patients and residential versus outpatients. The interaction effect of SARS-CoV-2 ± patients and gender on age was tested using an ANOVA model. The effects of gender, age group, residential status, and presence or absence of co-infections on SARS-CoV-2 ± rates were analyzed by chi-square. Prediction of SARS-CoV-2 + rates by age group, gender, number of co-infections, quantitative load of each co-infection and residential status was done by logistic regression. All analyses were performed using SAS™ (SAS Institute, Inc., Cary, NC, United States) statistical software.

## Results

### SARS-CoV-2 Assay

In SARS-CoV-2 + patients, the target were detected in the following frequency: ORF1ab, 99.8%, N Protein, 99.4%; and S Protein, 96.8%. There was 100% concordance between all three targets in all SARS-CoV-2 - patients.

### Study Population Description

The samples were collected from 12,075 subjects from 790 facilities throughout the United States. These consisted of doctor’s offices, medical clinics and hospitals, community health care centers, urgent care centers, assisted living facilities, retirement community centers and nursing homes. The average age of the population was 62.3 ± 24.4 years; and males (61.1 ± 24.4 years) and females (62.8 ± 24.2 years) were similar in age. SARS-CoV-2 + patients (72.4 ± 20.9) were 11.4 years older than SARS-CoV-2 - patients (60.6 ± 24.5) and this was statistically significant with *p* < 0.0001. Patients from nursing homes, hospice facilities, senior living facilities, long-term care facilities and rehab facilities were grouped together as residential (*N* = 4701, 38.9%). Healthcare or medical centers, urgent care facilities, community health centers, assisted living retirement communities were categorized as non-residential or outpatient (*N* = 7374, 61.1%). Residential patients were, on average, 13.1 years older than outpatients. The subject’s race was recorded only for 42% (*N* = 5,015) of subjects and the racial breakdown of the patients was 85.8% (*N* = 4,304) Caucasian, 12.4% (*N* = 619) African American, and 1.8% (*N* = 92) other races (Asian, Hawaiian and American Indians).

### SARS-CoV-2 and Demographic Comparison

Fourteen percent (*N* = 1690) of the samples were found to be SARS-CoV-2+. Of the 10816 of the subjects who reported their gender information, 13.4% of the males and 13.5% of the females tested as SARS-CoV-2+. Racial data was available only for 42% of the subjects. No differences in SARS-CoV-2 infection rates were observed between the different races, with 15.5% of Caucasian subjects and 12.6% of the African American subjects found to be SARS-CoV-2+. Of the 92 subjects from other races, 10% were found to be SARS-CoV-2+. Using chi-square tests it was found that gender and race had no effect on the SARS-CoV-2 infection rate. However, there were differences in SARS-CoV-2 infection rates between patients from residential facilities and outpatients, with 21.1% of residential subjects being SARS-CoV-2 + compared to 10% of the outpatient subjects (*p* < 0.0001). Also, the elderly group (age ≥62 years) had a SARS-CoV-2 infection rate of 18% compared to 8.4% in the younger patients (age < 62 years; *p* < 0.0001). Thus, race and gender had no effect on SARS-CoV-2 infection rate, while both residential status and age group had a significant effect on SARS-CoV-2 infection rate. The results are displayed in the Table 2.

### Other Pathogens Detected and Co-infection Rates

In the total population, patients also had other non-SARS-CoV-2 infections such as *S. aureus* (44.1%), Epstein-Barr Virus (HHV4) (23.0%), Human Herpes Virus 6 (HHV6) (25.7%), *M. catarrhalis* (14.1%), *K. pneumoniae* (2.2%), Human Metapneumovirus (hMPV) (2.7%), and Human Adenovirus (AdV) (1.0%). These data and the prevalence rates of co-infections in SARS-CoV-2 patients are listed below in Table 3. It was noted that 31% more patients had *S. aureus* in SARS-CoV-2 + group as compared to the SARS-CoV-2 – group. Similarly, HHV4, HHV6, *M. catarrhalis* and *K. pneumoniae* were found in 61, 39, 47, and 90% more patients in the SARS-CoV-2 + group compared to the SARS-CoV-2 – group, respectively. In contrast, significantly increased infection rates of hMPV and AdV were seen in SARS-CoV-2- patients as compared to SARS-CoV-2 + patients. The data are presented in Table 3.

**TABLE 3:** Infection rates for six respiratory pathogens in SARS-CoV-2 ± subjects.

**Pathogen**	**SARS-CoV-2 -/SARS-CoV-2 +**	**% Prevalence**			**Difference**	***p*-value**
		**All Subjects**	**SARS-CoV-2 −**	**SARS-CoV-2 +**	**%**	
*S. aureus*	4387/937	44.1	42.2	55.4	31	<0.0001
HHV4	2201/576	23.0	21.2	34.1	61	<0.0001
HHV6	2531/574	25.7	24.4	34.0	39	<0.0001
*M. catarrhalis*	1375/328	14.1	13.2	19.4	47	<0.0001
*K. pneumoniae*	205/64	2.2	2.0	3.8	90	<0.0001
hMPV	305/27	2.7	2.9	1.6	−45	0.0017
AdV	109/9	1.0	1.0	0.5	−50	0.04

Some of the subjects tested positive for more than one co-pathogen, with co-infection rates being greater in SARS-CoV-2 patients. In SARS-CoV-2 + patients, 86.3% had at least one co-infection compared to 75.7% in the SARS-CoV-2 – group (*p* < 0.0001). The degree of disparity between SARS-CoV-2 + and SARS-CoV-2 – groups increased with increasing number of co-infected pathogens. In patients who tested SARS-CoV-2+, 59% had two or more co-infections compared to 39.8% of the SARS-CoV-2- subjects (*p* < 0.0001). The percentage of subjects with three or more co-infections who were SARS-CoV-2 + (32.5%) was significantly greater than the percentage of SARS-CoV-2 – subjects with three or more co-infections (15.7%; *p* < 0.0001). Likewise, the percentage of SARS-CoV-2 + subjects with four or more co-infections (14.7%) was significantly greater than the percentage of SARS-CoV-2 – subjects with four or more co-infections (4.3%; *p* < 0.0001). This data is presented in Table 4. In further analysis of grouping of co-infections it was also noted that patients with HHV4 and HHV6 co-infections were twice as likely to be SARS-CoV-2 + (25.4%) than SARS-CoV-2 – (12.8%; *p* < 0.0001). SARS-CoV-2 + patients were more likely than SARS-CoV-2 – patients to have co-infections with HHV4 + *S. aureus*, HHV4 + *K. pneumoniae*, HHV6 + *S. aureus*, HHV6 + *K. pneumoniae*, or *S. aureus* + *K. pneumoniae* (*p* < 0.0001). Co-infection with *S. aureus* + *M. pneumoniae* was more prevalent in SARS-CoV-2 + versus SARS-CoV-2 – patients (*p* = 0.04). Analysis also revealed that SARS-CoV-2 + patients are more likely to have multiple co-infections, whereas the percentage of patients with a single co-infected pathogen is higher in the SARS-CoV-2 – (36.0%) versus SARS-CoV-2 + group (27.3%), and this difference was statistically significant (*p* < 0.0001).

**TABLE 4:** Number of co-infections in SARS-CoV-2 + vs SARS-CoV-2 -.

**Number of co-infections**	**SARS-CoV-2 +**		**SARS-CoV-2 −**	
	**N**	**%**	**N**	**%**
≥6	27	1.6	40	0.4
≥5	70	5.7	91	1.3
≥4	152	14.7	313	4.3
≥3	300	32.5	1188	15.7
≥2	448	59.0	2498	39.8
≥1	461	86.3	3735	75.7

### Logistic Regression Prediction of SARS-CoV-2 Positivity

A logistic regression model was used to predict SARS-CoV-2 positivity using other associate variables in the study. In the model, age group, gender and resident status were used as categorical variables, while log-transformed quantitative loads of HHV4, HHV6, *S. aureus*, *K. pneumoniae*, *M. catarrhalis*, and *M. pneumoniae* were used as continuous variables. The loads of the co-pathogen ranged from 10^1^ to 10^7^. The logistic model found that *M. pneumoniae* was not a predictor for SARS-CoV-2 + status, so it was removed from the model. It was found that all variables except gender are significantly associated with predicting SARS-CoV-2 + rates. The data is presented Table 5. Patients age 62 or older are more likely to be SARS-CoV-2 + than SARS-CoV-2 – [OR = 2.3, *p* < 0.0001, 95% confidence interval (2.01, 2.62)]. Similarly, residential patients (OR = 1.95, *p* < 0.0001), higher HHV4 viral load (OR = 1.17, *p* < 0.0001), higher HHV6 viral load (OR = 1.14, *p* < 0.0001), and higher bacterial loads of *S. aureus* (OR = 1.08, *p* < 0.0001), *K. pneumoniae* (OR = 1.12, *p* = 0.003) and *M. catarrhalis* (OR = 1.10, *p* < 0.0001) were significant predictors of SARS-CoV-2 + status. The lower confidence intervals for all variables were above 1. The use of logistic regression models to predict SARS-CoV-2 + status from the above-listed independent variables was supported by the higher model fit criteria of percent concordant statistic (68.5) and c statistic (0.69). The logistic regression models were repeated adding race into the model and found that race was not significantly associated with predicting SARS-CoV-2 + rate. Initially, Caucasian versus African American patients were examined, followed by Caucasian versus everyone else. Neither of the models displayed any significant race effect (results not presented).

**TABLE 5:** Logistic regression prediction of SARS-CoV-2 + status.

**Predictor Variable**	**Model test Statistics**	***p*-value**	**Odd Ratio**	**Lower CI**	**Upper CI**
Gender	0.52	0.47	1.05	0.93	1.18
AGE group	152.40	<0.0001	2.30	2.01	2.62
Resident Status	119.93	<0.0001	1.95	1.73	2.19
HHV4 Load	45.90	<0.0001	1.17	1.12	1.22
HHV6 Load	23.87	<0.0001	1.14	1.08	1.21
*S. aureus* Load	36.37	<0.0001	1.08	1.06	1.11
*K. pneumoniae* Load	8.68	0.003	1.12	1.04	1.21
*M. catarrhalis* Load	23.73	<0.0001	1.10	1.06	1.15

## Discussion

This is the first large-scale assessment of SARS-CoV-2 and co-infection rates with 38 other respiratory pathogens in a consecutive series of people presenting for SARS-CoV-2 testing during the COVID-19 pandemic in the United States. The major findings were that almost all patients have one or more co-pathogens, and that non-SARS-CoV-2 co-infection rates were significantly higher in SARS-CoV-2 + (86%) patients than SARS-CoV-2 – patients (76%) (*p* < 0.0001). *S. aureus*, Epstein-Barr Virus (HHV4), Human Herpes Virus 6 (HHV6), *M. catarrhalis*, and *K. pneumoniae* were the most common co-pathogens in SARS-CoV-2 + patients. In SARS-CoV-2- patients, the most common pathogens detected, in order of frequency, were *S. aureus*, Human Herpes Virus 6 (HHV6), Epstein-Barr Virus (HHV4), *M. catarrhalis*, hMPV, *K. pneumoniae* and AdV. The respiratory symptoms produced by these non-SARS-CoV-2 pathogens in 76% of SARS-CoV-2- patients may have been the reason for SARS-COV-2 testing, as the testing guidelines in place at the time were heavily weighted toward symptomatic patients. The PCR test used here, which tested for three different SARS-CoV-2 associated proteins, showed high reliability of the data that were used in this analysis.

### Clinical Significance of Co-pathogens

Some of the bacterial pathogens that were significantly increased in SARS-CoV-2 + compared to SARS-CoV-2 – patients (*S. aureus*, *K. pneumoniae*, *and M. catarrhalis*) can themselves compromise pulmonary function and cause pneumonia, especially in immunocompromised individuals (Henig and Kaye, 2017; Martin and Bachman, 2018). Previous studies have shown that respiratory viral infections predispose patients to bacterial infection with a more severe clinical course with greater morbidity and mortality (Prasso and Deng, 2017). Post-viral bacterial pneumonia produces complex interactions between the bacteria and viruses, normal nasopharyngeal bacterial flora, and the host immune system (Lee et al., 2016). For example, viral infection can damage the respiratory epithelium, enabling infiltration and infection by bacterial flora and subsequent pneumonia, and can also upregulate bacterial binding sites, facilitating infection (Lee et al., 2016). Additionally, viral infection can impair activity of monophages, a key participant in immune function (Lee et al., 2016). A characteristic of SARS-CoV-2 infections is significantly reduced lymphocyte counts (Li et al., 2020). Lymphocytes are essential for adaptive immunity. The very high levels of pro-inflammatory cytokines in SARS-CoV-2 “cytokine storm,” can cause lymphocyte apoptosis and resultant lymphocytopenia (Jensen et al., 2018). This lymphocytopenia constitutes an immunodeficient state, which could make these patients more susceptible to coinfection with other pathogens (Li et al., 2020).

In the present study, the observed rate of co-infection in SARS-CoV-2 + patients (86%) and SARS-CoV-2- patients (76%) was significantly higher than the rates observed in previous studies (0.6 to 50%). The higher coinfection rate observed in the present study may be due to the criteria applied to SARS-CoV-2 testing eligibility at the time, which was heavily weighted toward symptomatic patients, and whom would be more likely to have a current infection.

The samples were consecutive samples from a 4-week period beginning in the last week of March. Since that time, great differences in the rates of SARS-CoV-2 infection in different parts of the United States have evolved, and continue to evolve, most likely based on rates of opening of businesses and social venues, social distancing, use of masks, hand washing, etc. A repeat analysis of this kind, with the opportunity to recontact those who test positive for SARS-CoV-2 with and without infection with other pathogens known to produce serious respiratory symptoms, is under consideration. Such follow up data would be useful to determine the contribution of the co-infection to outcome.

Since the subjects in this study came from many parts of the United States, the findings reported here may be representative of the United States as whole at the time of sampling. Nevertheless, this was not an epidemiologic selected sample and may not reflect the true prevalence of the other pathogens. Thus, data from an epidemiologic sample is needed. The large number and diversity of subjects allowed for examination of SARS-CoV-2 and other pathogen infection rates, and co-infection by age group, residential facilities (i.e., nursing homes and assisted living facilities), race, and gender. As expected, co-infection rates were higher in elderly patients, however, young adults and some children were also co-infected. Rates of co-infection were similar in both Caucasians and African Americans, and males and females.

As with SARS-CoV-2, infection rates for common respiratory pathogens increased with age. The rate of SARS-CoV-2 infection in the >62 years age group was more than double that observed in younger patients. Co-pathogen rates were also greater in the elderly age group as a whole and even higher than those for SARS-CoV-2. Infection with non-SARS-CoV-2 pathogens was also greater in the SARS-CoV-2 – group in the elderly, indicating age-related vulnerability is a general phenomenon. It suggests that a negative test for SARS-CoV-2 in a symptomatic subject, particularly an elderly one, should be followed by testing for non- SARS-CoV-2 pathogens if such testing is not done during the testing for SARS-CoV-2.

Given how much more prevalent these non-SARS-CoV-2 respiratory infections are than SARS-CoV-2 itself, it may be worthwhile to also test for them in all elderly patients with moderate-severe respiratory symptoms. Absence of association with others infected with SARS-CoV-2 or a lack of travel to places with high rates of infected individuals supports testing for co-pathogens in the initial testing effort.

For the 76% of SARS-CoV-2 - patients co-infected with one or more respiratory pathogens, it is likely that respiratory symptoms in at least some are due, in part, to the co-infection, and the patient would benefit from treatment for that pathogen, e.g., *K. pneumoniae*. Co-pathogen testing can include testing for antibiotic resistance genes to aid in the selection of effective treatments for infections due to *S. aureus*, *K. pneumoniae* and *M. catarrhalis*, the most common serious co-pathogens detected in SARS-CoV-2 + patients in the present study.

In conclusion, we have utilized a highly reliable PCR-based test for SARS-CoV-2 and other respiratory pathogens, to provide the first countrywide data on the rate of infection with respiratory pathogens other than SARS-CoV-2. The infection rate for these other respiratory pathogens throughout the United States is much greater than that of SARS-CoV-2 itself in subjects seeking SARS-CoV-2 testing. Infection with these other respiratory pathogens as well as co-occurrence of infection with SARS-CoV-2 was increased in the elderly and those who reside in nursing homes. Gender and race did not impact rates of infection with other respiratory pathogens. These results suggest that patients with pre-existing respiratory infections are at increased risk to develop a SARS-CoV-2 infection, or conversely, that once infected with SARS-CoV-2, vulnerability to other respiratory pathogens is increased. Identifying and treating other respiratory pathogens along with SARS-CoV-2 testing should facilitate better outcomes in the current pandemic.

## Data Availability Statement

The raw data supporting the conclusions of this article will be made available by the authors, without undue reservation.

## Ethics Statement

Ethical review and approval was not required for the study on human participants in accordance with the local legislation and institutional requirements. Written informed consent to participate in this study was provided by the participants’ legal guardian/next of kin.

## Author Contributions

BM conceived and designed the study and was primary author the manuscript. KJ designed and conducted the statistical analyses. HM provided guidance and was secondary author of the manuscript. All authors contributed to the article and approved the submitted version.

## Conflict of Interest

HM receives grant support from Allergan Inc., Eli Lilly Inc., Janssen Pharmaceuticals, Quincy Biopharmaceuticals, and Sumitomo Dainippon. BM is an employee of Vikor Scientific LLC.

The remaining author declares that the research was conducted in the absence of any commercial or financial relationships that could be construed as a potential conflict of interest.
