# Supplementary material for: Respiratory Microbial Co-infection With SARS-CoV-2
Source: Front Microbiol. 2020 Aug 25;11:2079. doi: 10.3389/fmicb.2020.02079 (PMC7477285; doi:10.3389/fmicb.2020.02079)
Supplement: Supplementary file 1 [file Table_1.DOCX]

Supplementary Material

**Supplemental Table S1: SARS-CoV-2 status in subjects with two additional respiratory pathogens**

| **Variable** | **Category** | **SARS-CoV-2** | **SARS-CoV-2 + (%** | **SARS-CoV-2 N** | **SARS- CoV-2 (%)** | **Total N** | **p-value** |
| --- | --- | --- | --- | --- | --- | --- | --- |
| HHV4 + HHV6 | Both Yes | 301 | 25.4% | 882 | 74.6% | 1183 | P<0.0001 |
|  | No | 1389 | 12.8% | 9503 | 87.3% | 10892 |  |
| HHV4 + *S. aureus* | Both Yes | 401 | 24.5% | 1235 | 75.5% | 1636 | P<0.0001 |
|  | No | 1289 | 12.4% | 9150 | 87.7% | 10439 |  |
| HHV4 + *K. pneumoniae* | Both Yes | 37 | 35.6% | 67 | 64.4% | 104 | P<0.0001 |
|  | No | 1653 | 13.8% | 10318 | 86.2% | 11971 |  |
| HHV4 + *M. catarrhalis* | Both Yes | 162 | 27.5% | 427 | 72.5% | 589 | P<0.0001 |
|  | No | 1528 | 13.3% | 9958 | 86.7% | 11486 |  |
| HHV4 + *M. pneumoniae* | Both Yes | 10 | 21.7% | 36 | 78.3% | 46 | P=0.13 |
|  | No | 1680 | 14.0% | 10349 | 86.0% | 12029 |  |
| HHV6 + *S. aureus* | Both Yes | 384 | 21.8% | 1378 | 78.2% | 1762 | P<0.0001 |
|  | No | 1306 | 12.7% | 9007 | 87.3% | 10313 |  |
| HHV6 + *K. pneumoniae* | Both Yes | 28 | 27.5% | 74 | 72.5% | 102 | P<0.0001 |
|  | No | 1662 | 13.9% | 10311 | 86.1% | 11973 |  |
| HHV6 + *M. catarrhalis* | Both Yes | 162 | 26.0% | 462 | 74.0% | 624 | P<0.0001 |
|  | No | 1528 | 13.3% | 9923 | 86.7% | 11451 |  |
| HHV6 + *M. pneumoniae* | Both Yes | 11 | 19.6% | 45 | 80.4% | 56 | NS |
|  | No | 1679 | 14.0% | 10340 | 86.0% | 12019 |  |
| *S. aureus* + *K. pneumoniae* | Both Yes | 47 | 26.3% | 132 | 73.7% | 179 | P<0.0001 |
|  | No | 1643 | 13.8% | 10253 | 86.1% | 11896 |  |
| *S. aureus* + *M. catarrhalis* | Both Yes | 220 | 22.6% | 754 | 77.4% | 974 | P<0.0001 |
|  | No | 1470 | 13.2% | 9631 | 86.8% | 11101 |  |
| *S. aureus* + *M. pneumoniae* | Both Yes | 30 | 19.7% | 122 | 80.3% | 152 | P=0.04 |
|  | No | 1660 | 13.9% | 10263 | 86.1% | 11923 |  |
| *M. catarrhalis* +  *K. pneumoniae* | Both Yes | 15 | 28.8% | 37 | 71.2% | 52 | P=0.002 |
|  | No | 1675 | 13.9% | 10348 | 86.1% | 12023 |  |
| *M. catarrhalis* +  *M. pneumoniae* | Both Yes | 7 | 18.4% | 31 | 81.6% | 38 | NS |
|  | No | 1683 | 14.0% | 10354 | 86.0% | 12037 |  |

**Supplementary Table S2: Number of co-infections by SARS-CoV-2 status**

| **SARS-CoV-2 Positive** | | | **SARS-CoV-2 Negative** | | |
| --- | --- | --- | --- | --- | --- |
| **Number of co-infections** | **Number of subjects** | **%** | **Number of co-infections** | **Number of subjects** | **%** |
| 0 | 232 | 13.7% | 0 | 2520 | 24.3% |
| 1 | 461 | 27.3% | 1 | 3735 | 36.0% |
| 2 | 448 | 26.5% | 2 | 2498 | 24.1% |
| 3 | 300 | 17.8% | 3 | 1188 | 11.4% |
| 4 | 152 | 9.0% | 4 | 313 | 3.0% |
| 5 | 70 | 4.1% | 5 | 91 | 0.9% |
| 6 | 21 | 1.4% | 6 | 23 | 0.2% |
| 7 | 5 | 0.3% | 7 | 8 | 0.1% |
| 8 or more | 1 | 0.1% | 8 or more | 9 | 0.1% |

**Supplementary Table S3: Logistic regression prediction of SARS-CoV-2 + statu**s

| **Predictor Variable** | **Wald Chi-square test Statistics** | **p-value** | **OR**  **(Odd Ratio)** | **Lower CI for OR** | **Upper CI for OR** |
| --- | --- | --- | --- | --- | --- |
| Gender | 0.52 | 0.47 | 1.05 | 0.93 | 1.18 |
| AGE group | 152.40 | <.0001 | 2.30 | 2.01 | 2.62 |
| Resident Status | 119.93 | <.0001 | 1.95 | 1.73 | 2.19 |
| HHV4 Load | 45.90 | <.0001 | 1.17 | 1.12 | 1.22 |
| HHV6 Load | 23.87 | <.0001 | 1.14 | 1.08 | 1.21 |
| *S. aureus* Load | 36.37 | <.0001 | 1.08 | 1.06 | 1.11 |
| *K. pneumoniae* Load | 8.68 | 0.003 | 1.12 | 1.04 | 1.21 |
| *M. catarrhalis* Load | 23.73 | <.0001 | 1.10 | 1.06 | 1.15 |
